# Central nervous system infection: imaging findings suggestive of a fungus as the cause

**DOI:** 10.1590/0100-3984.2020.0093

**Published:** 2021

**Authors:** Lillian Gonçalves Campos, Thaylla Maybe Bedinot da Conceição, Marília Sfredo Krüger, Juliano Adams Perez, Juliana Ávila Duarte

**Affiliations:** 1 Hospital de Clínicas de Porto Alegre (HCPA), Porto Alegre, RS, Brazil.; 2 Medvia Diagnóstico, Porto Alegre, RS, Brazil.; 3 Hospital Moinhos de Vento, Porto Alegre, RS, Brazil.; 4 Hospital Municipal Getúlio Vargas, Porto Alegre, RS, Brazil.; 5 Universidade Federal do Rio Grande do Sul (UFRGS), Porto Alegre, RS, Brazil.

**Keywords:** Central nervous system fungal infections/diagnostic imaging, Mycoses/diagnosis, Brain abscess/diagnostic imaging, Tomography, X-ray computed, Magnetic resonance imaging, Infecções fúngicas do sistema nervoso central/diagnóstico por imagem, Micoses/diagnóstico, Abscesso encefálico/diagnóstico por imagem, Tomografia computadorizada, Ressonância magnética

## Abstract

Fungal infections of the central nervous system (CNS) are rare. However, because of the increase in the number of immunocompromised individuals, they have been gaining prominence in the differential diagnosis of CNS infections. Imaging techniques are sensitive for detecting and localizing an abnormality, in many cases allowing the origin of a lesion to be categorized as infectious, inflammatory, neoplastic, or vascular. This essay illustrates the magnetic resonance imaging and computed tomography findings of the most common fungal infections of the CNS, based on the experience of the Radiology Department of the Hospital de Clínicas de Porto Alegre, in the city of Porto Alegre, RS, Brazil.

## Abstract (pt)

As infecções fúngicas do sistema nervoso central (SNC) são raras, entretanto, com o aumento no número de indivíduos imunocomprometidos elas têm ganhado destaque no diagnóstico diferencial de infecções no SNC. As atuais técnicas de imagem são sensíveis para detectar uma anormalidade, localizá-la, e em muitos casos categorizar a lesão como de origem infecciosa e/ou inflamatória, neoplásica ou vascular. Este ensaio ilustra os achados de ressonância magnética e tomografia computadorizada mais comuns nas infecções fúngicas do SNC baseadas na experiência do Serviço de Radiologia do Hospital de Clínicas de Porto Alegre.

## INTRODUCTION

Fungal infections of the central nervous system (CNS) are rare. However, because of the increase in the number of immunocompromised individuals (those infected with HIV, those having undergone organ transplantation, and those having received chemotherapy) fungal infections of the CNS have been gaining prominence in the differential diagnosis of CNS infections^(1,2)^.

Fungal infections of the CNS can develop via hematogenous dissemination from a distant focus at a site such as the lung, through direct implantation after trauma, or from local extension of a sinonasal, orbital, or spinal infection^(1)^. Cerebrospinal fluid seeding has also been described as a less common cause of fungal infection of the CNS^(3)^.

Imaging techniques are sensitive for detecting and localizing abnormalities. In many cases, imaging can allow the origin of a lesion to be categorized as infectious, inflammatory, neoplastic, or vascular.

## IMAGING FINDINGS

### Cryptococcosis

**Figure 1:**
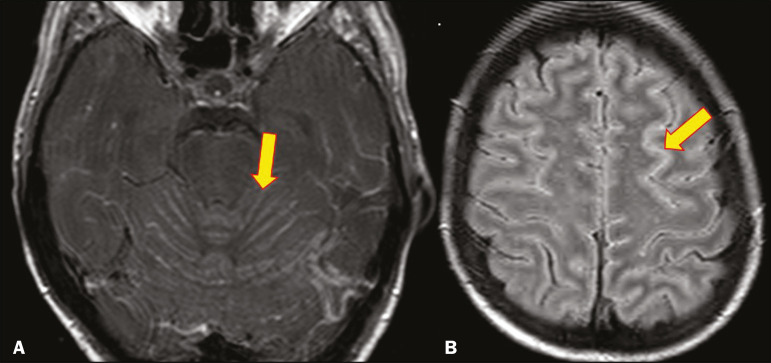
Cryptococcosis meningitis. **A**: Contrast-enhanced axial T1-weighted MRI sequence showing leptomeningeal enhancement (arrow). **B**: Axial FLAIR sequence showing leptomeningeal hyperintensity.

Infection with the encapsulated yeasts *Cryptococcus* spp., most commonly *Cryptococcus neoformans*, can result in meningitis or disseminated disease, especially in immunocompromised individuals^(4,5)^. The most common form of CNS involvement observed in patients with cryptococcosis is meningeal disease^(5)^. Although imaging findings are often lacking, some such patients show meningeal enhancement (Figure 1).

**Figure 2:**
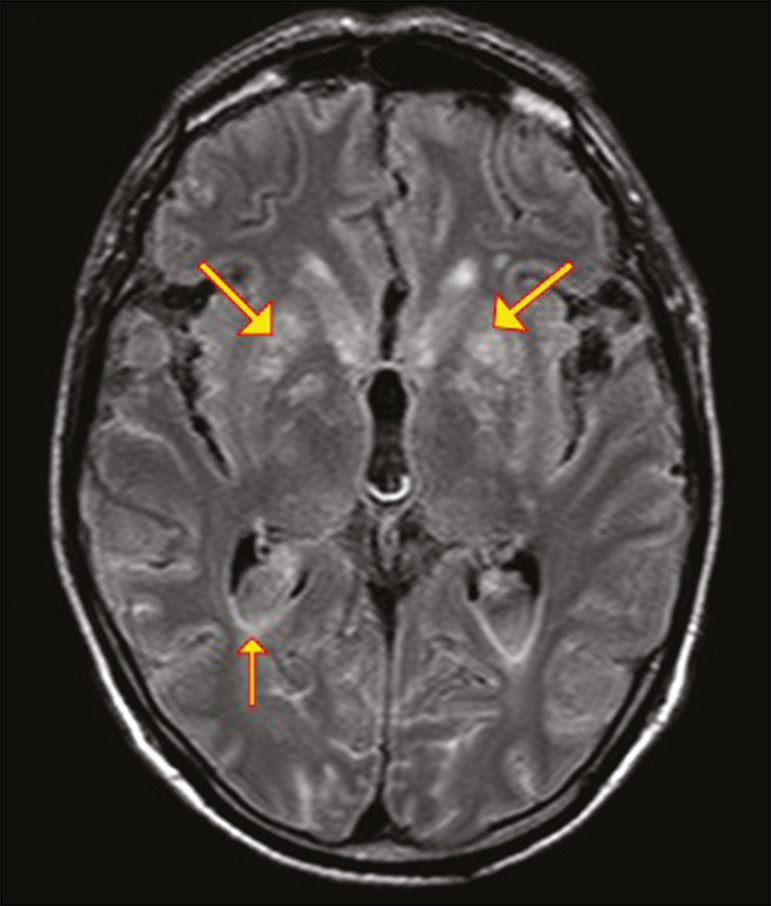
Axial FLAIR MRI sequence showing heterogeneous, predominantly hyperintense lesions in the basal nuclei, consistent with gelatinous pseudocysts (arrows), a classical finding of cryptococcosis of the CNS. Note also the granuloma in the choroid plexus (short arrow) and the hyperintense signal in the leptomeninges.

In patients with cryptococcosis, magnetic resonance imaging (MRI) can reveal dilated Virchow-Robin spaces with gelatinous cysts adjacent to the basal ganglia, secondary to the meningeal spread of CNS infection, as well as non-enhancing lesions, which are usually isointense to cerebrospinal fluid on T1- and T2-weighted images, although they can be slightly hyperintense on T1-weighted images (Figure 2) and, on diffusion-weighted imaging (DWI), may not show restricted diffusion^(3)^.

**Figure 3:**
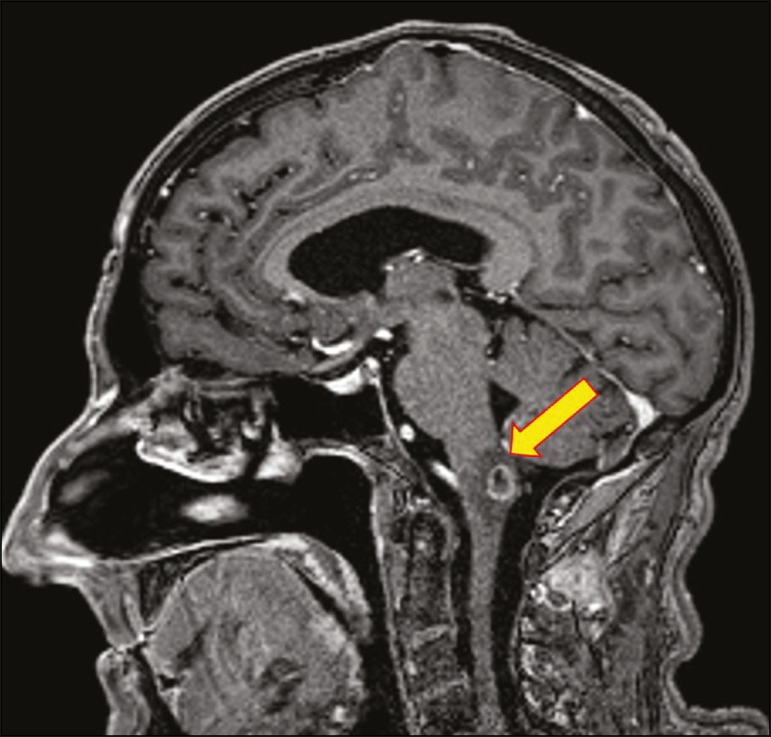
Cryptococcomas caused by *Cryptococcus gattii*. Gadolinium contrastenhanced sagittal T1-weighted MRI sequence showing a hypointense lesion with peripheral ring enhancement in the bulb (arrow), with signs of edema in the surrounding tissues.

In cases of cryptococcal meningitis, invasion of the adjacent brain parenchyma may give rise to cryptococcomas, which are chronic granulomas composed of lymphocytes, macrophages, and giant cells^(2)^. Cryptococcomas have been reported to demonstrate intermediate to low signal intensity on T1-weighted images and high signal intensity on T2-weighted images, with peripheral contrast enhancement (Figure 3) in some cases^(2,5)^. Most cryptococcomas are found in the basal ganglia or diffusely in the brain parenchyma^(2)^. In immunocompromised patients, these lesions rarely demonstrate enhancement after the administration of intravenous contrast^(2,3)^.

### Mucormycosis

Mucormycosis generally refers to infection with any fungi within the family Mucoraceae^(2)^. It usually affects patients who are immunocompromised^(6)^.

The imaging findings of cerebral mucormycosis are similar to those of aspergillosis, the most characteristic features including a propensity for sino-orbital disease and vascular invasion^(2)^. The computed tomography (CT) findings of sinonasal mucormycosis are sinus opacification, together with increased density or calcification/obliteration of the nasopharyngeal tissue planes^(6)^. The ethmoid sinus is the most commonly involved, the infection subsequently spreading via the valveless veins to the orbit, nose, cavernous sinuses, or brain parenchyma^(2)^. Bone destruction is a common finding.

**Figure 4:**
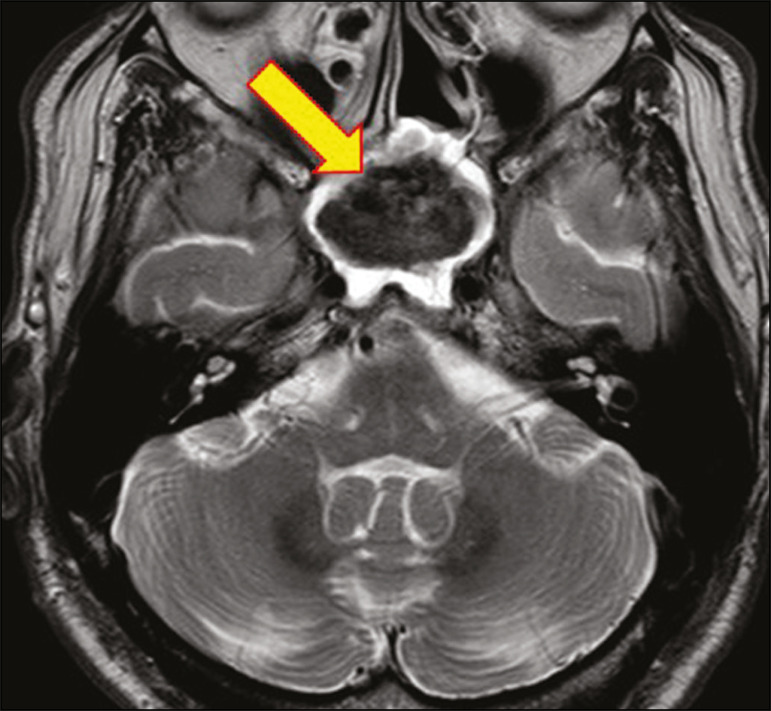
A 55-year-old patient with mucormycosis in the sphenoid sinus. Axial T2-weighted MRI sequence showing central hypointensity and a hyperintense halo.

**Figure 5:**
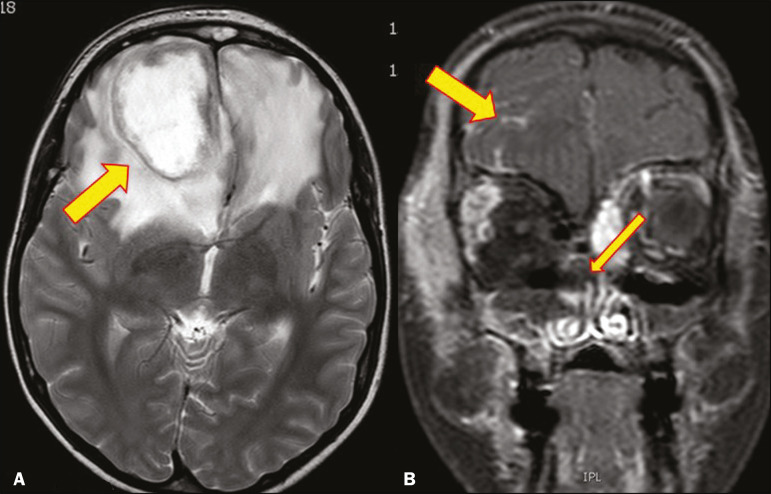
Mucormycosis with orbital and brain involvement. **A**: Axial T2-weighted MRI sequence showing a hyperintense lesion, a hypointense peripheral halo (arrow), and pronounced peripheral edema. **B**: Contrastenhanced coronal T1-weighted sequence showing a heterogeneous lesion in the right orbital region. Black turbinate sign (thin arrow), characterized by a lack of enhancement of the nasal turbinate mucosa. There is also leptomeningeal enhancement on the affected side (thick arrow).

Common MRI findings of mucormycosis include low signal intensity in the paranasal sinuses on T2-weighted fluid-attenuated inversion recovery (FLAIR) sequences (Figure 4), extension to the orbit with exophthalmos, thrombosis of the superior ophthalmic vein, extension through the orbital apex, and subsequent thrombosis of the cavernous sinus^(6)^. The infection can also extend into the infratemporal and pterygopalatine fossae from the maxillary sinus^(3)^. Loss of tissue enhancement is a strong sign of necrosis (Figure 5).

### Aspergillosis

Infection with *Aspergillus fumigatus* often leads to invasive tissue disease and is quite common in immunocompromised individuals. Primary aspergillosis of the paranasal sinus has been categorized as noninvasive (allergic fungal sinusitis) or invasive, and the dissemination can be direct or hematogenous^(7)^. The fungus has the propensity to spread along vessels that serve as direct channels for the seeding, with a tendency to invade the walls of small and large blood vessels^(7)^. Secondary complications of vascular invasion include vasculitis and infarction, as well as (more rarely) mycotic aneurysms and intracranial hemorrhage^(2)^.

In patients with aspergillosis, the CT findings are nonspecific for fungal disease, although findings such as a hyperdense mass in the sinuses with bony expansion or erosion are suggestive of the diagnosis^(2)^. When *A. fumigatus* reaches the intracranial compartment, brain parenchymal involvement is more common than is meningeal involvement^(2)^. Granuloma formation and/or encephalitis with or without abscess formation are possible manifestations.

**Figure 6:**
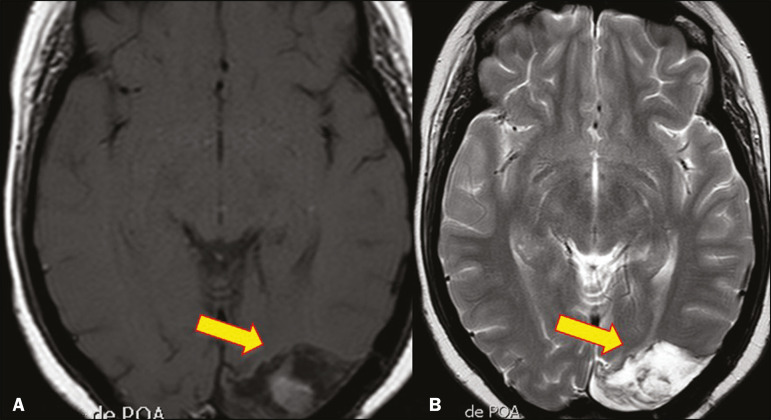
A 20-year-old patient with angioinvasive aspergillosis of the CNS. **A**: Axial T1-weighted MRI sequence showing a hyperintense aspergilloma, with a hypointense halo, in the left occipital region (arrow). **B**: Axial T2-weighted sequence showing a hyperintense lesion (arrow).

**Figure 7:**
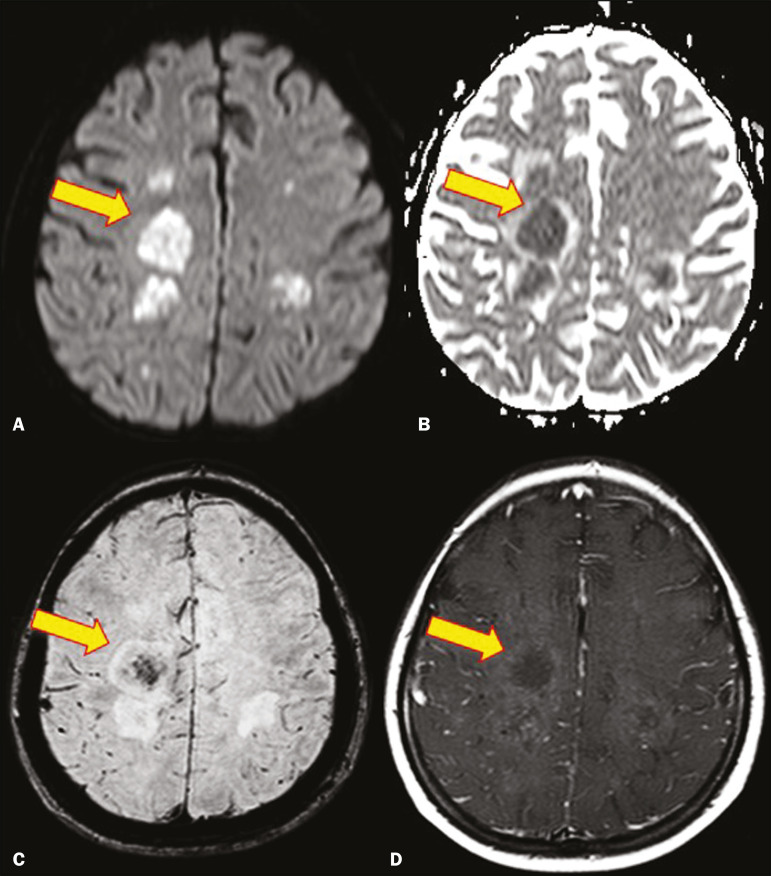
A 13-year-old immunocompromised patient with invasive aspergillosis of the CNS. **A,B**: Axial DWI and apparent diffusion coefficient map (arrows), respectively, showing restricted diffusion; **C**: Susceptibility-weighted imaging showing hemosiderin deposition (arrow); **D**: Contrastenhanced axial T1-weighted MRI sequence showing slight peripheral enhancement (arrow). Findings typical of fungal lesions caused by *Aspergillus* sp.

On MRI, abscesses caused by *Aspergillus* sp. may be surrounded by an area of low signal intensity on T2-weighted and gradient-echo images (Figure 6). Ring enhancement (a hypointense signal) on T2-weighted images has been attributed to small amount of hemorrhage with hemosiderin-laden macrophages and a dense population of hyphae at the rim of the abscess. As depicted in Figure 7, peripheral or central restricted diffusion may be seen on DWI^(2)^. Faint or no ring enhancement is most common and may be a diagnostic indicator (Figure 7). When present, rim enhancement can correlate with the formation of a capsule (granuloma) from chronic inflammation^(3)^.

### Histoplasmosis

**Figure 8:**
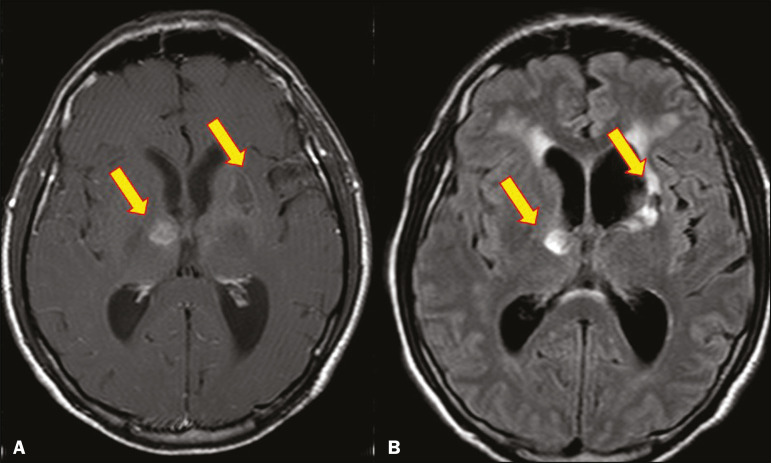
Infarcts by vasculitis secondary to histoplasmosis in a 38-yearold, previously healthy, patient who had lived in caves full of bats in Palestine and presented with progressive dementia. Biopsy demonstrated vasculitis secondary to histoplasmosis of the CNS. **A,B**: Contrast-enhanced FLAIR MRI sequences showing hyperintense lesions in the basal ganglia, consistent with subacute stroke.

Histoplasmosis is caused by the dimorphic fungus *Histoplasma capsulatum*. The most common manifestation of histoplasmosis is chronic meningitis involving the basal meninges, other presentations including acute meningitis, encephalitis, small ring-enhancing lesions, large brain abscesses, and stroke due to infected emboli or vasculitis^(2)^, as illustrated in Figure 8.

Lesions resulting from histoplasmosis, also known as “histoplasmomas”, tend to be small (< 2 cm) and round with peripheral ring enhancement. The lesions can be solitary but are more often multifocal and may occur in subcortical gray matter structures, as well as in the cerebellum, brainstem, or spinal cord. On MRI, such lesions typically show signals that are hypointense on T1-weighted images and variable (usually hypointense) on T2-weighted images, the latter thought to be attributable to the effects of paramagnetic contrast agents. On DWI, histoplasmomas may show variable signals depending on the presence of inflammatory cells and the type of necrosis^(3,8)^.

### Paracoccidioidomycosis

Paracoccidioidomycosis is caused by the fungus *Paracoccidioides brasiliensis* and is the most prevalent systemic mycosis among immunocompetent individuals in Latin America^(9,10)^. The initial infection occurs in the lungs, forming a primary pulmonary complex that may spread to other extrapulmonary foci by the lymphatic or hematogenous route. In up to 27% of cases of paracoccidioidomycosis, there is involvement of the CNS, a condition known as neuroparacoccidioidomycosis^(10)^.

Most paracoccidioidomycosis lesions are supratentorial, although infratentorial lesions are seen in some cases, and the lesions can be solitary or multiple. On CT, such lesions are typically hypodense, with ring or nodular enhancement after contrast injection, and are usually surrounded by mild edema^(9)^.

**Figure 9:**
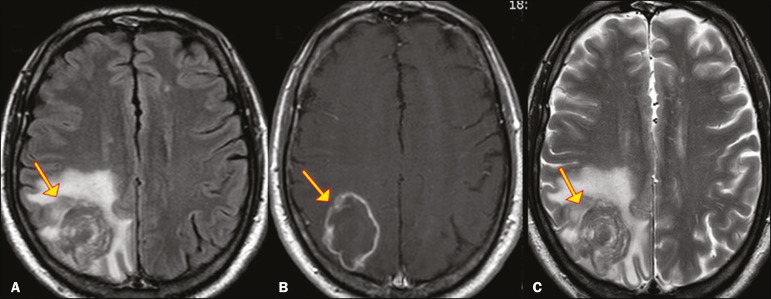
Neuroparacoccidioidomycosis. MRI sequences—FLAIR (**A**), contrast-enhanced axial T1-weighted (**B**), and axial T2-weighted (C)—identifying an expansile lesion in the right parietal lobe. On the FLAIR and T2-weighted images, the lesion showed a hypointense signal with peripheral enhancement and vasogenic edema surrounding the lesion, as well as having an onion-skin appearance on the T2-weighted images.

**Figure 10:**
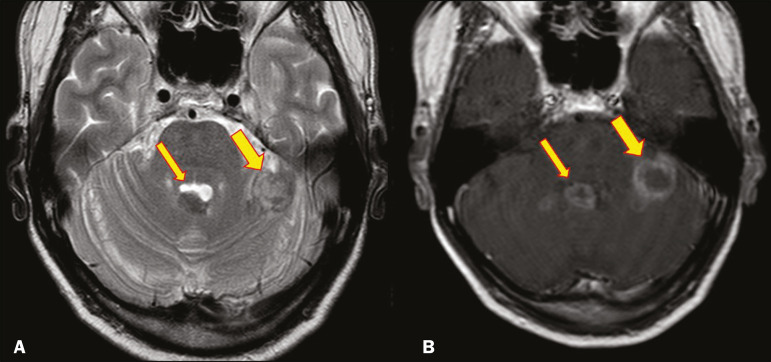
A 53-year-old alcoholic male patient, from a rural area, with a history of chewing grass. MRI showed granulomas in the CNS, suggestive of paracoccidioidomycosis. **A**: T2-weighted image showing lesions with heterogeneous low signal intensity in the cerebellum (arrows). **B**: Contrastenhanced T1-weighted image showing hypointense lesions with peripheral ring enhancement in the cerebellum (arrows).

In all patients with paracoccidioidomycosis, MRI shows a hyperintense signal at the periphery of the lesion on T1-weighted images. Paracoccidioidomycosis lesions typically show a hypointense signal on T2-weighted at the periphery, with an “onion-skin” appearance in some cases (Figure 9). Necrosis is also commonly observed, as is peripheral or heterogeneous contrast enhancement (Figure 10). A change in the perilesional white matter signal indicates vasogenic edema^(9,10)^. Although meningeal involvement is rare, it has been reported^(9)^.

### Cerebral candidiasis

Cerebral candidiasis normally results from infection with *Candida* sp., either systemic infection in immunocompromised patients or intravascular catheter infection^(2)^. The diagnosis does not usually pose a challenge, because patients typically develop microabscesses in the setting of known candidemia^(2)^. The CT findings are usually normal. On MRI, the microabscesses typically show ring enhancement and have a hemorrhagic component^(1,2)^.

## CONCLUSION

Radiologists play a central role in the diagnosis of major CNS infections. Although fungal infection of the CNS is rare, the diagnosis should be considered a possibility in certain clinical contexts. The most widely used imaging resources are MRI and CT. Whereas CT is more commonly used in situations of urgency (acute cases), in the search for focal lesions, and for the identification of complications such as hemorrhage and mass effect, MRI plays an important role in the elucidation of differential diagnoses because it has greater sensitivity in the specific characterization of each lesion. We can conclude that, in some cases, it is necessary to correlate imaging findings with clinical data, laboratory data, and biopsy findings in order to make the final diagnosis.

## References

[1] Gavito-Higuera J, Mullins CB, Ramos-Duran L (2016). Fungal infections of the central nervous system: a pictorial review. J Clin Imaging Sci.

[2] Mathur M, Johnson CE, Sze G (2012). Fungal infections of the central nervous system. Neuroimag Clin N Am.

[3] Starkey J, Moritani T, Kirby P (2014). MRI of CNS fungal infections: review of aspergillosis to histoplasmosis and everything in between. Clin Neuroradiol.

[4] Qazzafi Z, Thiruchunapalli D, Birkenhead D (2007). Invasive Cryptococcus neoformans infection in an asplenic patient. J Infect.

[5] Chrétien F, Lortholary O, Kansau I (2002). Pathogenesis of cerebral Cryptococcus neoformans infection after fungemia. J Infect Dis.

[6] Petrikkos G, Skiada A, Lortholary O (2012). Epidemiology and clinical manifestations of mucormycosis. Clin Infect Dis.

[7] Boes B, Bashir R, Boes C (1994). Central nervous system aspergillosis. Analysis of 26 patients. J Neuroimaging.

[8] Nguyen FN, Kar JK, Zakaria A (2013). Isolated central nervous system histoplasmosis presenting with isquemic pontine stroke and meningitis in an immune-competent patient. JAMA Neurol.

[9] Reis F, Collier PP, Souza TF (2013). Neuroparacoccidioidomycosis (NPCM): magnetic resonance imaging (MRI) findings. Mycopathologia.

[10] Almeida SM (2005). Central nervous system paracoccidioidomycosis: an overview. Braz J Infect Dis.

